# Supplementary material for: Improved Equine Fecal Microbiome Characterization Using Target Enrichment by Hybridization Capture
Source: Animals (Basel). 2024 Jan 29;14(3):445. doi: 10.3390/ani14030445 (PMC10854943; doi:10.3390/ani14030445)
Supplement: Supplementary file 1 [file animals-14-00445-s001.zip › animals-2788708-supplementary.pdf]

SUPPLEMENTARY MATERIALS

Table S1. Counts of reads, OTUs and Good's coverage index per sample.

	Sample 1			Sample 2			Sample 3			Sample 4		
	TEHC	TEHC Normalized	16S-Amplicon	TEHC	TEHC Normalized	16S-Amplicon	TEHC	TEHC Normalized	16S-Amplicon	TEHC	TEHC Normalized	16S-Amplicon
Raw reads	1,144,928	-	563,114	727,571	-	564,542	1,235,085	-	508,936	1,044,957	-	697,643
Filtered reads	847,125	475,231	475,231	531,323	489,748	489,748	945,679	446,346	446,346	797,226	601,577	601,577
16S mapping reads	-	300235	323506	-	315252	350801	-	316794	354840	-	380515	389125
% Reads mapped	-	63	68	-	64	72	-	71	79	-	63	65
OTUs	-	11046	4981	-	10058	5340	-	9032	4036	-	10178	4641
Good's coverage index	-	0.9932619	0.9944545	-	0.9945789	0.9944612	-	0.9934879	0.995412	-	0.9949805	0.9954616

	BEI MOCK MICROBIOME		
	TEHC	TEHC Normalized	16S-Amplicon
Raw reads	5,345,638	-	136,184
Filtered reads	4,591,609	56,879	56,879
16S mapping reads	-	38,427	42,261
% Reads mapped	-	68	74
OTUs	-	1,940	634
Good's coverage index	-	97.73857	99.29722

Table S2. Relative abundance (%) of genera identified among the most abundant exclusively with one sequencing strategy.

Fecal Sample	Genus	TEHC	16S-AMP
1	*Phascolarctobacterium*	0.99	1.85
	Pseudobutyrivibrio	0.72	1.55
2	*Fibrobacter*	1.47	0.92
	BF311	0.77	1.62
	Solibacillus	0.99	1.26
3	*Blautia*	1.37	0.95
	Lysinibacillus	**8.43**	**0.17**
	Parabacteroides	0.95	1.29
	Streptococcus	1.01	0.86
4	*Clostridium*	1.17	0.68
	Lysinibacillus	**1.45**	**0.04**
	Paludibacter	0.98	1.82
	Phascolarctobacterium	0.95	1.46
	Wautersiella	0.90	1.11
	YRC22	0.42	1.37

Table S3. Relative abundance (%) of species identified among the most abundant exclusively with one sequencing strategy.

Fecal Sample	Genus	TEHC	16S-AMP
1	*F. succinogenes*	2.12600	2.90505
2	*F. succinogenes*	1.44710	0.91590
	C. variabile	0.85551	1.27850
	S. luteciae	5.88386	6.65819
3	***L. boronitolerans***	**8.41399**	**0.17191**
	B. muralis	0.94383	1.11318
4	*B. uniformis*	1.40628	2.39404
	A. lwoffii	**1.41308**	**0.00026**
	L. boronitolerans	**1.44751**	**0.00694**

Table S4. Relative abundance (%) of expected species identified with 16S-amplicon sequencing and TEHC.

Species	16S-Amplicon	TEHC	Expected
Helicobacter pylori	12.19	5.96	5
Neisseria cinerea	7.17	4.38	5
Staphyloccocus aureus	5.81	4.03	5
Staphylococcus epidermidis	4.28	2.88	5
Propionibacterium acnes	0.36	3.72	5
Rhodobacter sphaeroides	2.49	2.56	5
